# Structural basis of inactivation of Ras and Rap1 small GTPases by Ras/Rap1-specific endopeptidase from the sepsis-causing pathogen *Vibrio vulnificus*

**DOI:** 10.1074/jbc.RA118.004857

**Published:** 2018-10-03

**Authors:** Song Yee Jang, Jungwon Hwang, Byoung Sik Kim, Eun-Young Lee, Byung-Ha Oh, Myung Hee Kim

**Affiliations:** From the ‡ Department of Biological Sciences, Korea Advanced Institute of Science and Technology, Daejeon 34141,; the § Infection and Immunity Research Laboratory, Metabolic Regulation Research Center, Korea Research Institute of Bioscience and Biotechnology, Daejeon 34141, and; the ¶ Department of Food Science and Engineering, Ewha Womans University, Seoul 03760, Korea

**Keywords:** toxin, virulence factor, sepsis, host–pathogen interaction, bacterial pathogenesis, effector, MARTX toxin, Ras/Rap1-specific endopeptidase, Vibrio vulnificus

## Abstract

Multifunctional autoprocessing repeats-in-toxin (MARTX) toxins are secreted by Gram-negative bacteria and function as primary virulence-promoting macromolecules that deliver multiple cytopathic and cytotoxic effector domains into the host cytoplasm. Among these effectors, Ras/Rap1-specific endopeptidase (RRSP) catalyzes the sequence-specific cleavage of the Switch I region of the cellular substrates Ras and Rap1 that are crucial for host innate immune defenses during infection. To dissect the molecular basis underpinning RRSP-mediated substrate inactivation, we determined the crystal structure of an RRSP from the sepsis-causing bacterial pathogen *Vibrio vulnificus* (*Vv*RRSP). Structural and biochemical analyses revealed that *Vv*RRSP is a metal-independent TIKI family endopeptidase composed of an N-terminal membrane-localization and substrate-recruitment domain (N lobe) connected via an inter-lobe linker to the C-terminal active site–coordinating core β-sheet–containing domain (C lobe). Structure-based mutagenesis identified the 2His/2Glu catalytic residues in the core catalytic domain that are shared with other TIKI family enzymes and that are essential for Ras processing. *In vitro* KRas cleavage assays disclosed that deleting the N lobe in *Vv*RRSP causes complete loss of enzymatic activity. Endogenous Ras cleavage assays combined with confocal microscopy analysis of HEK293T cells indicated that the N lobe functions both in membrane localization via the first α-helix and in substrate assimilation by altering the functional conformation of the C lobe to facilitate recruitment of cellular substrates. Collectively, these results indicate that RRSP is a critical virulence factor that robustly inactivates Ras and Rap1 and augments the pathogenicity of invading bacteria via the combined effects of its N and C lobes.

## Introduction

Numerous Gram-negative bacterial pathogens have the ability to deliver bacterial “effector” proteins into the host cytoplasm to modulate a multitude of host cell functions (1). Direct effector delivery systems, known as type III (2, 3), IV (4, 5), and VI (6, 7) secretion systems, play a central role in pathogenic interactions between bacteria and host cells. In addition to the typical effector transfer systems, many Gram-negative pathogenic bacteria, including the sepsis-causing pathogen *Vibrio vulnificus*, secrete multifunctional autoprocessing repeats-in-toxin (MARTX)^5^ toxins via the atypical type 1 secretion system (8, 9). Once secreted, these toxins translocate their effector domains modularly into host cells and undergo autoproteolysis, resulting in the release of functionally discrete effectors in the cytosol (10, 11). MARTX toxins are believed to form pores in the host cell plasma membrane via repeat-containing regions located at the N and C termini that facilitate effector module translocation, although how the repeat regions form pores remains unknown (10–12). Autoproteolysis essential for MARTX toxin function is mediated by a core element of the toxins, namely the cysteine protease domain that is allosterically activated by inositol hexakisphosphate exclusively present in the host cytosol (13).

Within host cells, different effectors display distinct cytopathicity or cytotoxicity, and the overall toxicity depends on the combined virulence of individual MARTX toxin effectors (12, 14). Because pathogenic bacteria confer variation on the effector domain content of MARTX toxins by homologous recombination events, MARTX toxin-mediated translocation of novel effector domains emerges spontaneously (15, 16). Thus, characterizing the cellular functions of individual effector domains can provide a comprehensive understanding of how MARTX toxins contribute to bacterial pathogenesis.

To date, more than 10 MARTX effector domains have been annotated, including several domains of unknown function in *Vibrio* species (17), among which the fifth domain of a MARTX toxin from *Vibrio vulnificus* CMCP6 is a Ras/Rap1-specific endopeptidase (RRSP) (18). Notably, bacterial strains that produce a MARTX toxin harboring an RRSP are more virulent than those that do not (15). RRSP catalyzes sequence-specific cleavage of the Switch I region of the cellular substrates Ras and Rap1 (18, 19) that are critical for a myriad of signal transduction events, including innate immune defenses (20, 21).

Structural and functional bioinformatics and cell biological studies revealed that RRSP belongs to the TIKI superfamily (22), and it is composed of an N-terminal region mediating its localization to the plasma membrane (23) and a C-terminal region responsible for cytotoxicity (24). The N-terminal region, also referred to as the membrane localization domain (MLD), is conserved in various bacterial toxins (23, 25). Structural analysis of MLD alone revealed a bundle-shaped domain composed of four helices (26). The cytotoxicity of the RRSP from *V. vulnificus* CMCP6 depends on the C-terminal subdomain (residues 3672–3855) harboring residues Asp-3721 and Arg-3841 that are critical for maintaining the structural stability of the RRSP (24). Although these results revealed that RRSP hydrolyzes the critical cellular substrates Ras and Rap1, the molecular mechanisms by which it recognizes and processes cellular substrates remain unknown.

In this study, we determined the crystal structure of an RRSP from the sepsis-causing pathogen *V. vulnificus* CMCP6 (*Vv*RRSP), and we demonstrated that it is a metal-independent TIKI family endopeptidase. We further dissected the structural features, identified catalytic and substrate recognition residues, and unveiled the roles of the subdomains in the functions of the enzyme.

## Results

### Crystal structure of VvRRSP

**Figure 1.**
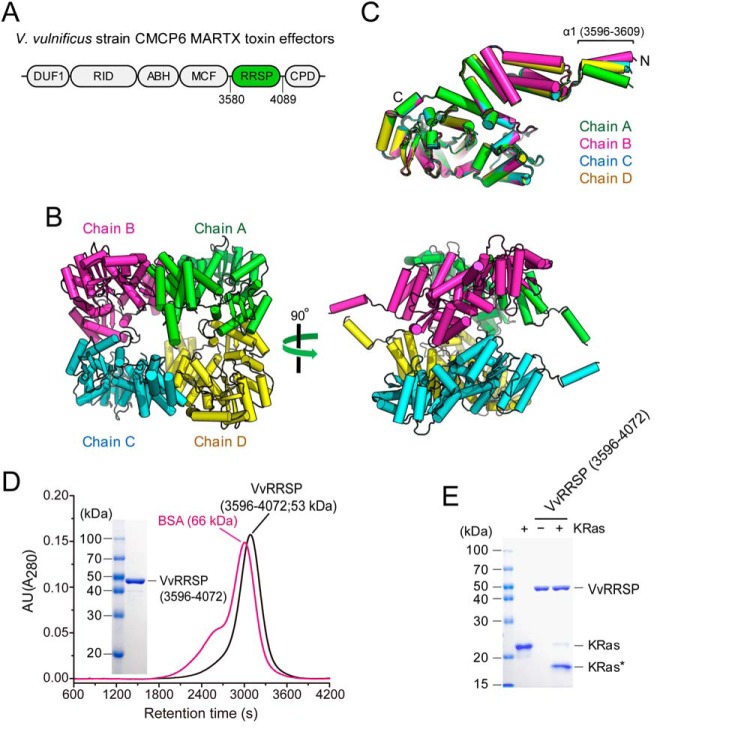
**Overall structure of the MARTX toxin effector RRSP from *V. vulnificus* CMCP6.** *A*, schematic representation of MARTX effector domains of *V. vulnificus* CMCP6, of which *Vv*RRSP (residues 3580–4089) is shown in *green. B*, four *Vv*RRSP molecules in the asymmetric unit (chains A–D) are colored *green, magenta, cyan,* and *yellow,* respectively. *C*, superimposition of *Vv*RRSP molecules in the asymmetric unit. *D*, size-exclusion chromatographic analysis of purified *Vv*RRSP (residues 3596–4072) in *black*, with bovine serum albumin (*BSA*; *magenta*) used as a size marker. *E*, *in vitro* KRas cleavage assay of purified *Vv*RRSP used for structure determination. KRas* indicates KRas cleaved by *Vv*RRSP.

A previous study reported that the MARTX toxin effector RRSP is an enzyme possessing site-specific processing activity against the small GTPases Ras and Rap1 that act as cellular switches during signal transduction, including innate immune responses (18). Computational protein sequence analysis revealed that RRSP belongs to the TIKI superfamily (22). To elucidate the structural basis of Ras and Rap1 processing by RRSP, we attempted crystallization of the full-length domain, corresponding to amino acid residues 3580–4089 of the MARTX toxin from *V. vulnificus* strain CMCP6 (Fig. 1*A*). However, the resulting crystals failed to diffract to high resolution. Deletion of N- and C-terminal flexible regions resulted in a core construct (residues 3596–4072) that produced crystals diffracting X-rays to 2.7 Å (Table 1), with four *Vv*RRSP molecules in the asymmetric unit (Fig. 1*B*; see under “Experimental procedures” for detailed information on structure determination and refinement). The structure of each of the four protein chains is essentially identical, except for the N-terminal α1 helix spanning residues 3596–3609 (Fig. 1*C*). Size-exclusion chromatography revealed that *Vv*RRSP exists as a monomer in solution (Fig. 1*D*); hence, the structure of chain A was considered representative of the subunit structure and was used in further analysis. The enzyme activity of *Vv*RRSP (residues 3596–4072) was confirmed by *in vitro* KRas cleavage assay, demonstrating that the construct was functional and processed Ras and Rap1 (Fig. 1*E*).

**Table 1:** **X-ray data collection and refinement statistics for RRSP and RRSP_E3930L/H4030L_**

	SeMet-RRSP	RRSP	RRSP_E3930L/H4030L_
**Data collection**			
Space group	C2221	C2221	C2221
Cell dimensions			
*a, b, c* (Å)	177.78, 198.18, 165.00	177.69, 197.93, 163.29	178.54, 198.28, 164.02
α, β, γ (°)	90, 90, 90	90, 90, 90	90, 90, 90
Wavelength	0.97940	0.97960	0.97960
Resolution (Å)	50–2.90 (2.95–2.90)*^a^*	50–2.70 (2.71–2.70)	50–2.30 (2.34–2.30)
No. of total reflections	859,157	135,922	840,598
No. of unique reflections	63,955 (3177)	73,652 (6166)	128,159 (6340)
Redundancy	13.4 (13.8)	6.6 (4.2)	6.6 (6.5)
Completeness (%)	100 (100)	94.52 (80.30)	99.9 (100)
*I*/σ*I*	18.7 (2.0)	17.27 (4.56)	19.87 (4.6)
*R*_pim_	0.474 (0.058)	0.247 (0.064)	0.285 (0.049)
*R*_meas_	0.214 (1.542)	0.173 (0.521)	0.126 (0.736)

**Refinement**			
Resolution		31.71 − 2.71 (2.81 − 2.71)	34.88 − 2.30 (2.38 − 2.30)
*R*_work_/*R*_free_*^b^*		0.23/0.27	0.18/0.23
Root mean square deviation			
Bond lengths		0.002	0.008
Bond angles		0.51	1.038
No. of atoms			
Proteins		14584	14,576
Ligands		61	45
Waters		245	1099
Average *B*-factors			
Proteins		42.70	45.10
Ligands		72.10	82.20
Waters		24.50	45.50
Geometry (%)			
Favored region		96.18	98.00
Allowed region		3.55	1.84
Outliers		0.27	0.16

*^a^* Numbers in parentheses are values for the highest resolution shell.

*^b^ R*_work_ = Σ‖*F*_obs_| − |*F*_calc_‖/Σ|*F*_obs_|. *R*_free_ = Σ‖*F*_obs_| − |*F*_calc_‖/Σ|*F*_obs_|, where all reflections belong to a test set of randomly selected data.

### Molecular details of VvRRSP

**Figure 2.**
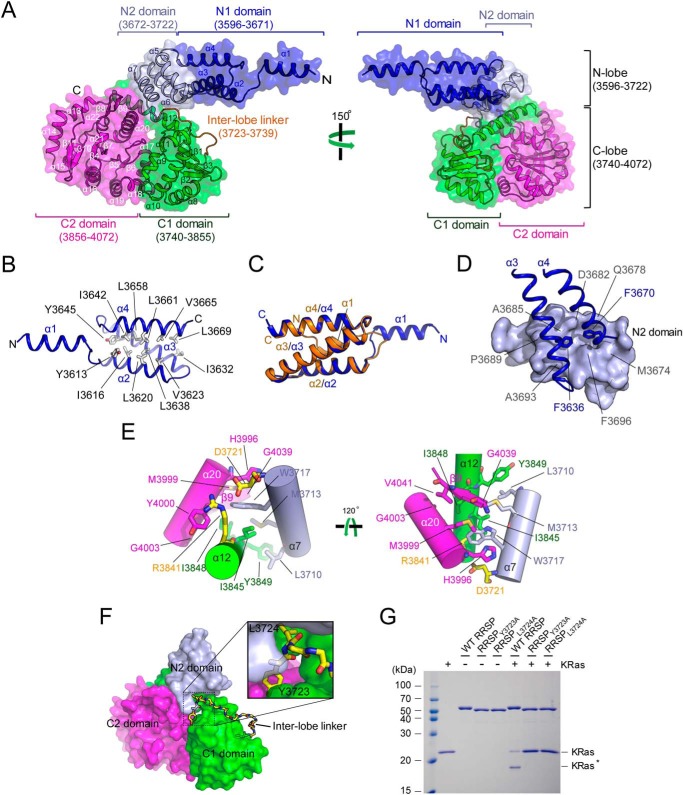
**Molecular details of *Vv*RRSP.** *A*, structure of *Vv*RRSP divided into subdomains. The N1, N2, C1, and C2 domains of *Vv*RRSP are colored *blue, gray, green*, and *magenta*, respectively. The linker region between the N and C lobes (inter-lobe linker) is colored *orange. B*, three-helix bundle within the N1 domain. Residues forming the hydrophobic core of the bundle are indicated. *C*, superimposition of the *Vv*RRSP N1 domain (*blue*) onto the *PMT* (PDB code 2EBF) C1 domain (*orange*). *D*, structural stabilization of the N1 domain. The critical residues Phe-3636 of α3 and Phe-3670 of α4 that are involved in structural stabilization are indicated. Residues involved in forming the groove in the N2 domain are shown in *light blue* surface representation and are indicated. *E*, structural integration between the C lobe and the N2 domain. Helix α20 in the C2 domain (*magenta*), α12 in the C1 domain (*green*), and α7 in the N2 domain (*light blue*) are displayed in *cartoon* representation. Residues involved in hydrophobic and charge interactions between the domains are indicated. *F*, inter-lobe linker connecting the two lobes shown in *stick* representation. The N2 domain in the N lobe and the C1 and C2 domains in the C lobe are colored *light blue, green,* and *magenta*, respectively. The *enlarged box* shows residues Tyr-3723 and Leu-3724 that may be critical for controlling flexibility between the N and C lobes. *G*, *in vitro* KRas cleavage assays showing the importance of residues Tyr-3723 and Leu-3724. KRas* indicates cleaved KRas.

The *Vv*RRSP structure can be divided into an N-terminal lobe (N lobe, residues 3596–3722) and a C-terminal lobe (C lobe, residues 3740–4072; Fig. 2*A*), and the N lobe can be further subdivided into the membrane localization N1 domain (MLD), consisting of four helices (α1–α4), as characterized previously (23), and the N2 domain comprising three helices (α5-α7). The MLD resembles a pointing finger in which the first α-helix extends from the remaining three-helix bundle (Fig. 2*A* and Fig. S1*A*). This bundle (α2, α3, and α4) forms a hydrophobic core, in which Tyr-3613, Ile-3616, Leu-3620, Val-3623, Ile-3632, Leu-3638, Ile-3642, Tyr-3645, Leu-3658, Leu-3661, Val-3665, and Leu-3669 are arranged to maintain structural integrity (Fig. 2*B*). The structural arrangement of the MLD in *Vv*RRSP is markedly different from that of the two structures of isolated MLDs determined previously by nuclear magnetic resonance (NMR; PDB code 2N9W; Fig. S1*B*) (26) and crystallography (PDB code 4ERR; Fig. S1*C*), suggesting that this domain may be highly flexible. The *Vv*RRSP MLD is similar to the membrane localization C1 domain in the *Pasteurella multocida* toxin (PMT) (23), in which the first α-helix of the MLD differs but the remaining three helices superimpose well in the two protein structures (Fig. 2*C*). Residues Phe-3636 on the α3 helix and Phe-3670 on the α4 helix make critical contributions to the structural stabilization of the MLD by engaging in extensive hydrophobic contacts with the groove lined by Met-3674, Gln-3678, Asp-3682, Ala-3685, Pro-3689, Ala-3693, and Phe-3696 on the α5 and α6 helices of the N2 domain (Fig. 2*D*).

The C lobe can also be separated into two subdomains (Fig. 2*A*), the C1 domain (α8–α12 and β1–β3), and the C2 domain (α13–α22 and β4–β11) that adopts a typical α + β TIKI fold (Fig. S2*A*). The C lobe is firmly integrated with the N lobe, mainly via hydrophobic interactions between Leu-3710, Met-3713, and Trp-3717 on α7 in the N2 domain; Ile-3845, Ile-3848, and Tyr-3849 on α12 in the C1 domain; His-3996, Met-3999, Tyr-4000, and Gly-4003 on α20; and Gly-4039 and Val-4041 on β9 in the C2 domain (Fig. 2*E*). A previous study reported that Asp-3721 and Arg-3841 are critical for structural stability of RRSP (24). The structure of RRSP reveals that Asp-3721 on α7 forms a salt bridge with Arg-3841 on α12 that helps to maintain the structure between N2 and C1 domains (Fig. 2*E*).

A long loop (residues 3723–3739, referred to as the inter-lobe linker) connects the two lobes (Fig. 2*F*). In particular, residues Tyr-3723 and Leu-3724 play a critical role in maintaining structural integrity between the N2, C1, and C2 domains (Fig. 2*F*). The inter-lobe linker appears to function as a hinge to bestow flexibility between the two lobes. To evaluate the importance of the loop, residues Tyr-3723 and Leu-3724 were mutated to alanine, and *in vitro* KRas cleavage assays revealed that RRSP enzyme activity was completely abolished (Fig. 2*G*). Thus, these two residues might be essential for controlling flexibility.

Structural comparison using PDBeFold identified structural similarity between *Vv*RRSP and TIKI superfamily proteins (22), including PMT (PDB code 2EBF; RMSD = 2.79 Å for 404 Cα atoms), HopBA1 (PDB code 5T09; RMSD = 2.96 Å for 146 Cα atoms), Bcr135 (PDB code 3B55; RMSD = 2.47 Å for 140 Cα atoms), and ChaN (PDB code 2G5G; RMSD = 2.95 Å for 145 Cα atoms), all of which contain a parallel β-sheet core surrounded by α-helices (Fig. S2).

### C2 catalytic domain

TIKI superfamily proteins share a common core domain consisting of a central β-sheet surrounded by α-helices (22). Likewise, the C2 domain in *Vv*RRSP has a six-stranded β-sheet, flanked by helices on both sides (Fig. S2*A*). Five of the six β-strands of the C2 domain (β4, β5, β6, β7, and β10) form the characteristic parallel β-sheet core observed in all TIKI proteins (Fig. S2, *B–F*).

**Figure 3.**
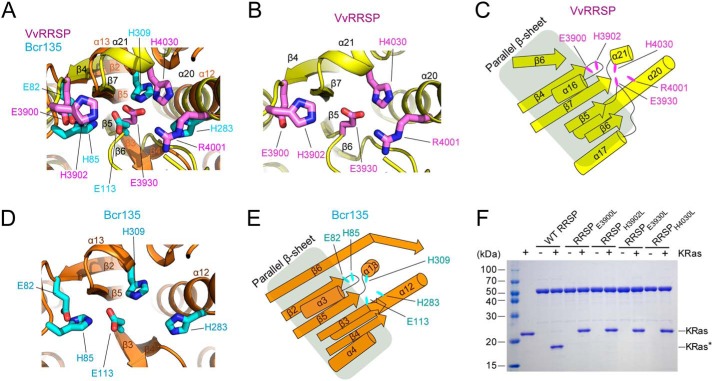
**C2 catalytic domain.** *A–E*, structural comparison of *Vv*RRSP with the Ere family protein Bcr135 (PDB code 3B55). *A*, superimposition of *Vv*RRSP onto Bcr135. *A*, *B*, and *D*, structures of the *Vv*RRSP C2 domain and Bcr135 are colored *yellow* and *orange*, respectively. Functional residues of *Vv*RRSP and Bcr135 are colored *magenta* and *cyan*, respectively. *C* and *E*, topologies of the parallel β-sheet core and neighboring helices of the *Vv*RRSP C2 domain and Bcr135 colored *yellow* and *orange*, respectively. Functional residues are shown as *elliptical shapes* in *magenta* for RRSP and *cyan* for Bcr135. *F*, effects of *Vv*RRSP catalytic residues on KRas cleavage in vitro. KRas* indicates cleaved KRas.

It has been proposed that enzymatically active TIKI family proteins share a catalytic mechanism with the erythromycin esterase (Ere) family, in which a conserved His–Glu pair is essential for substrate hydrolysis (27). Structural comparison of *Vv*RRSP with the Ere family protein Bcr135 revealed that functional residues are highly conserved (Fig. 3, *A–E*). The predicted functional residues His-3902, His-4030, Glu-3900, and Glu-3930 (forming the 2His/2Glu motif) in *Vv*RRSP are located near β4, β5, and β7 strands within the parallel β-sheet core of the C2 domain (Fig. 3, *B* and *C*, and Fig. S3). We subsequently investigated whether these 2His/2Glu residues are essential for hydrolysis of Ras and Rap1 by mutating them to leucine. *In vitro* KRas cleavage assays demonstrated that none of RRSP_H3902L_, RRSP_H4030L_, RRSP_E3900L_, or RRSP_E3930L_ catalyzed the proteolytic cleavage of KRas, indicating that all four 2His/2Glu residues are essential for catalytic activity (Fig. 3*F*). Mutant proteins did not undergo any significant conformational changes compared with WT RRSP according to the results of circular dichroism (CD) spectrometry analysis (Fig. S4).

**Figure 4.**
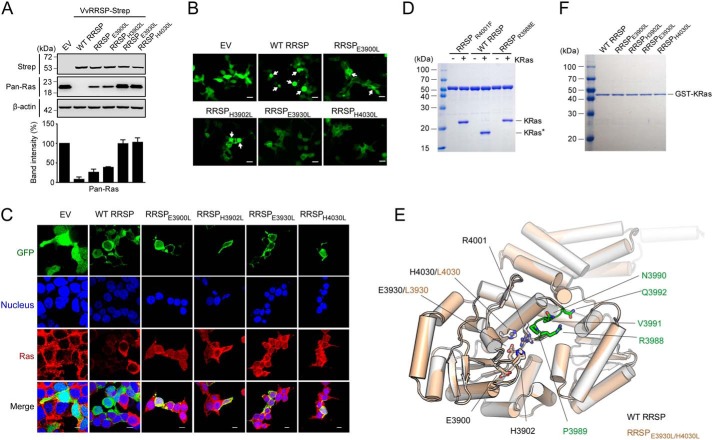
**Essential residues of the C2 catalytic domain for hydrolysis of endogenous Ras.** *A*, analysis of *Vv*RRSP catalytic residues in endogenous Ras cleavage. HEK293T cells transfected with plasmids expressing the indicated Strep-tagged proteins were lysed and immunoblotted with anti-Strep or anti-Ras (*Pan-Ras*) antibodies. Actin was used as a loading control. *EV*, empty vector. Data are representative of at least three independent experiments with similar results, and values are expressed as mean ± S.D. *B*, morphological differences of HEK293T cells transfected with GFP fusion plasmids expressing the indicated RRSP proteins. *White arrows* indicate severely shrunken cells. *C*, confocal microscopy analysis of WT and mutant RRSPs during cleavage of endogenous Ras. HEK293T cells were transfected with the indicated RRSP plasmids expressing GFP fusion proteins. After 16 h, cells were stained with anti-Pan-Ras antibody (*red*), and nuclei were stained with Hoechst (*blue*). *Scale bars* = 10 μm. *D*, analysis of *Vv*RRSP residues involved in substrate recognition during KRas cleavage *in vitro. E*, superimposition of the RRSP_E3930L/H4030L_ mutant structure (*light orange*) onto the WT RRSP structure (*white*). Functional residues are shown in *stick* representation. Residues located in the disordered region in the WT RRSP structure are indicated in *stick* representation (*green*) in the structure of mutant RRSP. *F*, pulldown assays of RRSP proteins using GST-fused KRas.

We further evaluated the importance of the 2His/2Glu residues for the hydrolysis of endogenous Ras (Fig. 4*A*) or Rap1 (Fig. S5) in HEK293T cells transfected with each *Vv*RRSP mutant. The results clearly showed that mutation of Glu-3930 or His-4030 completely abolished enzymatic activity, consistent with the results of *in vitro* KRas cleavage assays. Interestingly, mutation of Glu-3900 or His-3902 inhibited enzymatic activity significantly, but not completely, in cells (Fig. 4*A*). Consequently, ectopic expression of RRSP_E3930L_ or RRSP_H4030L_ for 16 h did not cause the cell-rounding that was observed with wildtype (WT) RRSP (Fig. 4*B*), as typically observed in RRSP-expressing cells (24). Mild cell-rounding was observed following RRSP_E3900L_ or RRSP_H3902L_ expression (Fig. 4*B*).

Green fluorescent protein (GFP)-fused MLD of RRSP localizes to the plasma membrane in HeLa cells (23), and we expected similar localization for *Vv*RRSP in this study. We therefore tested whether the *Vv*RRSP mutations affected cellular localization, and no difference was found between WT and mutant proteins at 16 h post-transfection; all proteins were mainly localized to the HEK293T cell membrane, where their substrates reside (Fig. S6). We further evaluated the ability of the mutants to cleave endogenous Ras at 16 h post-transfection by confocal microscopy analysis, and WT RRSP efficiently processed endogenous Ras, whereas mutants lacking essential catalytic residues were inactive (Fig. 4*C*). Thus, these results collectively demonstrate that *Vv*RRSP is a Ras/Rap1-specific endopeptidase that requires the essential 2His/2Glu catalytic residues in the C2 domain.

RRSP cleaves between residues Tyr-32 and Asp-33 in the Switch I region (^30^DEYDPTIEDSY^40^ in K/H/N-Ras, and ^30^EKYDPTIEDSY^40^ in Rap1A/B) of the cellular substrates Ras and Rap1 following sequence-specific substrate recognition (19). Remarkably, the sequences in the Switch I region are dominated by several negatively charged residues (Fig. S7) that might be involved in forming contacts with the RRSP active site. In addition to the catalytic His/Glu residues, a highly conserved basic residue is found near the active site residues in all TIKI proteases (22). The corresponding residue in *Vv*RRSP is Arg-4001 (Fig. 3, *B* and *C*). We therefore presumed that Arg-4001 may be a substrate recognition residue. Proteolytic processing assays with RRSP mutated at Arg-4001 revealed complete loss of catalytic activity, indicating that this residue may indeed play a role in substrate recognition (Fig. 4*D*).

During refinement of the RRSP structure, we noticed that the electron density map around residues Arg-3988–Gln-3992 located close to the active site in the C2 domain is disordered (Fig. 4*E*), indicating flexibility and a potential involvement in substrate recognition. Given that the catalytic residues of *Vv*RRSP were successfully assigned, we hypothesized that substitution of catalytic residues with nonfunctional residues might stabilize the active site and the adjacent flexible region. We subsequently determined the structure of the RRSP_E3930L/H4030L_ mutant at a resolution of 2.3 Å (Table 1). The structure of the mutant RRSP is essentially identical to that of WT RRSP (RMSD of Cα atoms is less than 0.5 Å), with minimal differences in the conformation of the active sites containing the essential residues (Fig. 4*E*). Like WT RRSP, the active site of the *Vv*RRSP_E3930L/H4030L_ mutant is arranged similarly to that of BcR135 (Fig. S8). Luckily, the flexible region spanning residues Arg-3988–Gln-3992 in molecule A of the mutant is ordered and adopts a loop structure close to the active site (Fig. 4*E*). To evaluate the involvement of this region in the function of RRSP, we selected Arg-3988 based on its interactions with the Switch I region of Ras, dominated by negatively charged residues (Fig. S7). This residue was mutated to glutamate, and the overall conformation was confirmed by CD spectroscopy (Fig. S4). *In vitro* KRas cleavage assays showed that the mutation completely inhibited enzymatic activity (Fig. 4*D*), suggesting that the flexible loop may be involved in substrate recognition.

Because we could not observe an interaction between WT RRSP and Ras, we used the inactive RRSP mutant to examine interactions with KRas. However, *in vitro* pulldown assays revealed that the nonfunctional RRSP was similarly unable to maintain interaction with KRas (Fig. 4*F* and Fig. S9). No differences in the active sites were observed between WT and mutant RRSP structures. Therefore, these results suggest that residues Glu-3900, Glu-3930, His-3902, and His-4030 in the active site may spontaneously adopt the catalytically active conformation in the presence of substrate.

Comparison of *Vv*RRSP and homologs revealed the highest similarity with the C1/C2 domains of PMT (PDB code 2EBF; RMSD = 2.96 Å for 404 Cα atoms; Fig. S2), even though *Vv*RRSP shares only 26% amino acid sequence identity with PMT. PMT also belongs to the TIKI superfamily, but it lacks the catalytic 2His/2Glu motif in the common β-sheet core (Fig. S10, *A* and *B*). Accordingly, endopeptidase activity against KRas was not observed for the PMT C1/C2 domain (Fig. S10*C*).

### VvRRSP is a metal-independent TIKI family endopeptidase

**Figure 5.**
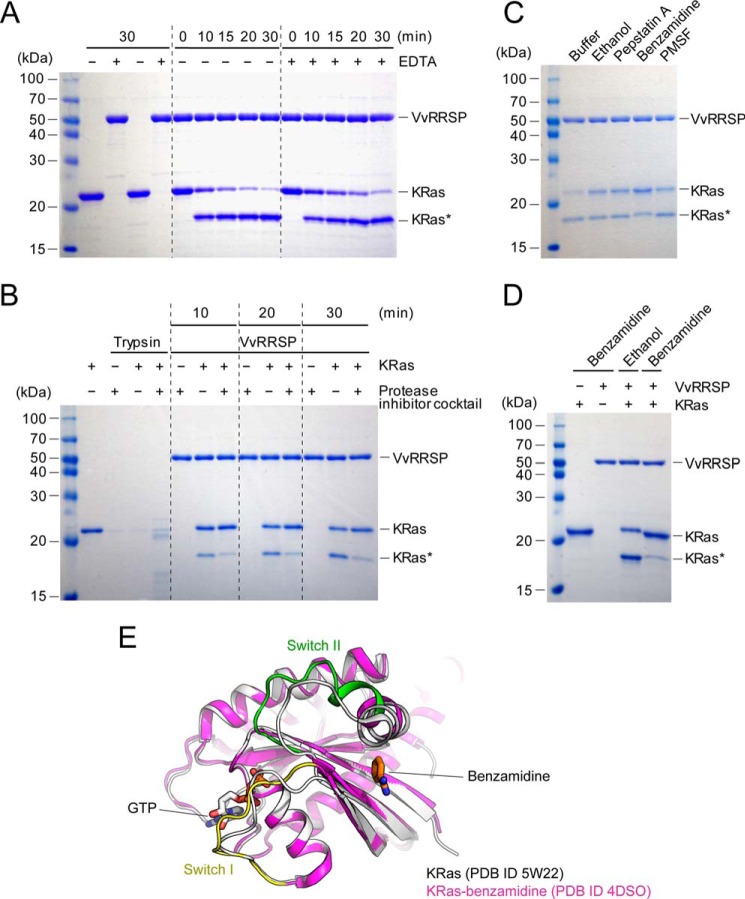
**VvRRSP is a metal-independent Ras-specific endopeptidase.** *A*, *in vitro* KRas cleavage assays showing metal-independent RRSP activity. KRas* indicates cleaved KRas. *B*, effects of protease inhibitor cocktails including phenylmethanesulfonyl fluoride, pepstatin, leupeptin, benzamidine, and bestatin on the KRas cleavage activity of RRSP. Trypsin was used as a control. *C*, *in vitro* KRas cleavage assay showing that benzamidine inhibits the activity of RRSP. Ethanol was used as a control. *D*, effect of benzamidine (4 mm) on the KRas cleavage activity of RRSP. Data are representative of two independent experiments with similar results. *E*, superimposition of the structure of KRas complexed with GTP (PDB 5W22, *white*) onto that of KRas complexed with GTP and benzamidine (PDB 4DSO, *magenta*). Switch I and II regions in the structure of KRas complexed with GTP and benzamidine are colored *yellow* and *green*, respectively. Benzamidine and GTP are shown in *stick* representation.

Tiki, involved in the Wnt signaling pathway, is a representative TIKI protease with metal ion-dependent activity (28). However, studies on EreB and Bcr136 showed that metal ions are required for structure formation of the enzymes, rather than enzymatic activity (27). Although we did not observe any metal bound to *Vv*RRSP during refinement of its structure, we biochemically examined whether it belongs to the TIKI metalloprotease family. The enzymatic activity of *Vv*RRSP toward KRas was comparable in the presence or absence of EDTA (Fig. 5*A*), indicating that metal ions are not involved in RRSP function. We also searched for other inhibitors that might be associated with blocking the enzymatic activity of RRSP, and a protease inhibitor mixture solution inhibited enzymatic activity of *Vv*RRSP (Fig. 5*B*). Subsequent analysis of the separate components revealed that benzamidine was responsible for inhibition of KRas cleavage by *Vv*RRSP (Fig. 5, *C* and *D*). It is reported that KRas directly interacts with benzamidine (Fig. 5*E*) (29), suggesting that the inhibitory effect may be induced by the binding of benzamidine to KRas, rather than RRSP. We therefore performed isothermal titration calorimetry, and no direct interaction was detected between *Vv*RRSP and benzamidine (data not shown). Together, these results indicate that *Vv*RRSP is a metal-independent TIKI family endopeptidase.

### Role of the VvRRSP N lobe

**Figure 6.**
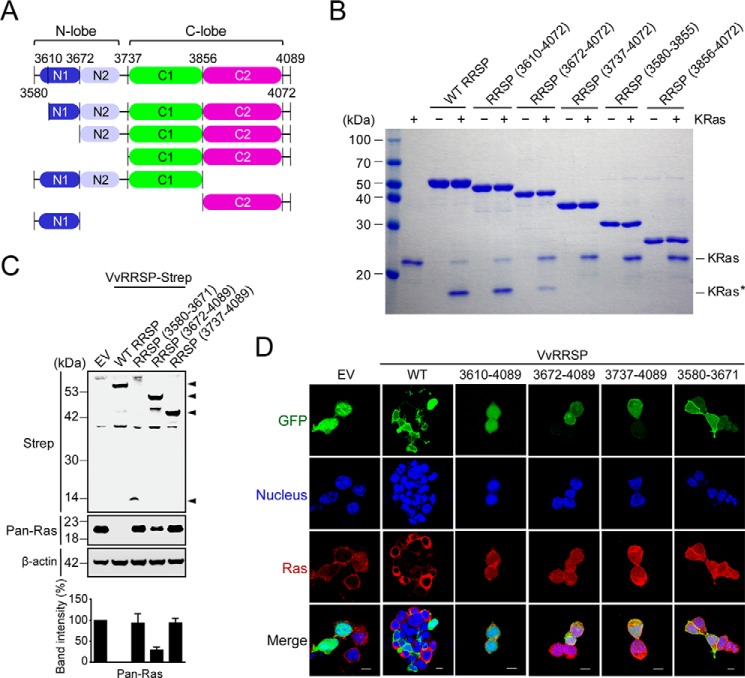
**Roles of the N lobe in *Vv*RRSP.** *A*, schematic representation of *Vv*RRSP consisting of the four domains N1 (*blue*), N2 (*light blue*), C1 (*green*), and C2 (*magenta*). *B*, *in vitro* KRas cleavage assay of RRSP domains. KRas* indicates cleaved KRas. *C*, effects of the N domain on endogenous Ras cleavage. HEK293T cells transfected with plasmids expressing the indicated Strep-tagged proteins were lysed and immunoblotted with anti-Strep or anti-Ras (*Pan-Ras*) antibodies. *Arrows* indicate RRSP expression bands. Actin was used as a loading control. *EV*, empty vector. Data are representative of at least three independent experiments with similar results, and values are expressed as mean ± S.D. *D*, roles of the N domain in localization and enzymatic activity of RRSP in cells. HEK293T cells were transfected with the indicated RRSP constructs expressing GFP fusions. After 16 h, cells were stained with anti-Pan-Ras antibody (*red*), and nuclei were stained with Hoechst (*blue*). *Scale bars* = 10 μm.

TIKI proteases have a wide variety of domains appended to the conserved TIKI fold structure that might act to promote enzymatic function (30). *Vv*RRSP has an N-terminally extended N lobe as part of its MLD. Thus, to further assess the role of the N lobe, we constructed various deletion mutants (Fig. 6*A*) and evaluated them by *in vitro* KRas cleavage assay (Fig. 6*B*). Deletion of the N lobe and inter-lobe linker (Δ3580–3736), leaving only the C lobe, resulted in complete loss of enzymatic activity toward KRas (Fig. 6*B*). Similarly, the C2 catalytic domain alone (residues 3856–4072) was unable to hydrolyze KRas (Fig. 6*B*). Interestingly, deletion of the MLD N1 domain (Δ3580–3671) significantly reduced the efficiency of KRas cleavage activity compared with WT RRSP (Fig. 6*B*). Thus, these results suggest that the N lobe and inter-lobe linker are crucial for substrate recruitment or recognition via integration with the C lobe to induce the functionally competent conformation of *Vv*RRSP. It is worth mentioning that the inter-lobe linker may act as a flexible hinge to properly arrange the two lobes into a functional conformation that is enzymatically active. In particular, Tyr-3723 and Leu-3724 on the linker may be key residues regulating flexibility (Fig. 2, *F* and *G*). Only deletion of α1 (Δ3580–3609) within the MLD failed to affect the enzymatic activity (Fig. 6*B*).

Consistent with the *in vitro* assay results, the cleavage of endogenous Ras was entirely blocked in HEK293T cells transfected with plasmid expressing the N lobe deletion mutant (residues 3737–4089), and the activity of the MLD-deleted RRSP (residues 3672–4089) was significantly impaired (Fig. 6*C*). We further analyzed the cellular localization and enzymatic activity of *Vv*RRSP by confocal microscopy. As reported previously (23), the MLD alone (residues 3580–3671) is mainly localized to the membrane in HEK293T cells that do not exhibit Ras cleavage activity when ectopically expressing this truncated construct (Fig. 6*D*). However, the MLD-deletion mutant (residues 3672–4089) or the C lobe mutant (residues 3737–4089) was dominantly expressed in the cytoplasm, again without enzymatic activity (Fig. 6*D*). Remarkably, the α1 helix appears to be critical for membrane localization of RRSP. Without the α1 helix (Δ3580–3609), membrane localization does not occur, resulting in reduced enzymatic activity, indicating that proper membrane localization of RRSP is required for enzymatic function (Fig. 6*D*). It is worth noting that in the structure the α1 helix points away from the MLD helix bundle (Fig. 2*A*). Taken together, these results demonstrate that the N lobe of RRSP performs dual roles: localization to the membrane via the α1 helix of the MLD and promoting enzymatic function.

## Discussion

Although mainly studied in the context of broad medical research in tumor initiation, progression, and metastasis, Ras proteins also play fundamental roles in immunity and inflammation (20). The Ras superfamily of small GTPases can be divided into at least six subfamilies (Ras, Rho, Ran, Rab, Rheb, and ARF). Among these, Ras subfamily proteins K/H/N-Ras and Rap1A/B have an identical amino acid sequence in their switch loops (18). This feature may confer the specificity of RRSP toward these two cellular substrates. The activation of Ras-dependent extracellular signal-regulated kinase-1 (ERK) signaling plays crucial roles in T-cell responses and Fcγ receptor-mediated phagocytosis (31–33). ERK signaling is also related to Toll-like receptor-mediated chemokine production in dendritic cells (34). GTP-bound Ras activates the phosphoinositide 3-kinase pathway that is crucial for the activation and functioning of all immune cells (32, 35, 36). Rap1 is also implicated in the activation of the innate immune system in various immune cells (37–40). Thus, it can be anticipated that robust inactivation of Ras and Rap1 by RRSP would lead to the failure of innate immune defenses and consequent enhancement of the pathogenicity of invading bacteria.

Bacterial effectors use diverse mechanisms to modulate the activity of host small GTPases and interfere with corresponding host-signaling pathways. Many effectors delivered by type III and IV secretion systems modulate the GTPase cycle of proteins belonging to the Rho, Rab, and ARF families by mimicking the functions of host regulators (41). Effectors also modulate the activity of small GTPases via modifications such as adenylation, glucosylation, *N*-acetylglucosamination, and proteolytic cleavage (42–46). Thus, bacterial effectors preferentially target molecular switch GTPases to efficiently subvert diverse cellular responses in the host–pathogen interactions.

The RRSP effector from the highly virulent *V. vulnificus* strain CMCP6 clinical isolate was initially identified as a MARTX toxin effector that is cytotoxic in HeLa cells (24), and it was later confirmed as a site-specific endopeptidase that effectively processes Ras and Rap1 (18). However, a lack of structural information has impeded our understanding of the mechanisms underlying how it recognizes and processes its cellular substrates. Our current structural analysis reveals that *Vv*RRSP shares an active site architecture with TIKI superfamily proteins (22), including Ere family enzymes known to catalyze the hydrolysis of esters and proteins (27). It has been proposed that enzymatically active TIKI domain proteins share a catalytic mechanism with Ere family enzymes, wherein the His–Glu pair conserved among TIKI domain proteins activates a water molecule for nucleophilic attack on the carbonyl group of ester and peptide bonds (22, 27). His-85 and Glu-113 form the His–Glu pair in Bcr135, corresponding to His-3902 and Glu-3930 in *Vv*RRSP (Fig. 3*A*). It remains uncertain whether *Vv*RRSP shares the same catalytic mechanism with TIKI proteins because the His-4030–Glu-3930 pair was assigned as the essential catalytic residues in *Vv*RRSP, rather than the His-3902–Glu-3930 pair (Fig. 4, *A* and *B*). However, we cannot rule out the possibility that *Vv*RRSP shares a catalytic mechanism with TIKI proteins because a water molecule was observed close to Glu-3900 and Glu-3930 in WT *Vv*RRSP (Fig. S11*A*) and in the center of the active site of the catalytically nonfunctional mutant (Fig. S11*B*).

The structure of *Vv*RRSP revealed that the functional conformation of the N1 domain is promoted by the N2 domain (Fig. 2*D*). This structural feature may render the 14-amino acid residue α1 helix suitable for membrane anchoring (Fig. 2*A*). One side of α1 is composed of hydrophobic residues, and the other side has positively charged residues that may enhance membrane interaction (Fig. S12*A*). Superimposition of *Vv*RRSP molecules in the asymmetric unit reveals different conformations for the MLD fingers (Fig. 1*C*), indicating flexibility. Indeed, part of the α1 helix is involved in crystal packing contacts, its flexibility is independent of the rest of the helices of the N1 domain in the RRSP structure, and it may be stabilized by these contacts (Fig. S12*B*). Thus, we cannot rule out the possibility that the α1 helix conformation in the structure of *Vv*RRSP may be due to the crystal packing contacts. The membrane-interacting conformation of the N lobe likely stabilizes the functional conformation of RRSP in association with the active site-containing C lobe, thereby promoting recruitment of substrates from the membrane.

Pathogenic bacteria have evolved mechanisms to effectively combat host defenses. Expanding our understanding of how bacteria manipulate immune systems in humans will provide promising strategies for controlling infection. Targeting the Ras and Rap1 signaling switches could offer an efficient way to subvert host signaling systems and augment the virulence of pathogenic bacteria. Because RRSPs are conserved among pathogens infecting mammals, fish, and plants, our detailed structural and functional analysis of *Vv*RRSP might be broadly applicable for the development of strategies to combat powerful virulence mechanisms.

## Experimental procedures

### Bacterial strains, plasmids, and cell culture

Bacterial strains and plasmids used in this study are listed in Table S1. *Escherichia coli* strains were grown in Luria-Bertani (LB) medium at 37 °C with appropriate antibiotics. HEK293T cells were purchased from the American Type Culture Collection (ATCC) and cultured at 37 °C with 5% CO_2_ in Hyclone Dulbecco's modified Eagle's medium (GE Healthcare) supplemented with 1% antibiotic/antimycotic (Gibco-BRL) and 10% fetal bovine serum (Gibco-BRL).

### DNA cloning and mutagenesis

Genomic DNA from *V. vulnificus* strain CMCP6 was kindly provided by Dr. Joon Haeng Rhee from Chonnam National University Medical School and utilized as a template for amplifying the DNA fragment encoding the effector domain (residues 3580–4072) of the MARTX toxin, named *Vv*RRSP. The amplified DNA fragment was subcloned into the NcoI and XhoI restriction enzyme sites of the pHis-Parallel1 expression vector, a protein expression vector that attaches an N-terminal His_6_-tag cleavable using recombinant tobacco etch virus (rTEV) protease. To investigate the critical residues responsible for endopeptidase activity, constructs for expression of RRSP_E3900L_, RRSP_E3930L_, RRSP_H3902L_, RRSP_H4030L_, RRSP_Y3723A_, RRSP_L3724A_, RRSP_R3988E_, and RRSP_R4001F_ were generated using site-directed mutagenesis. For protein structural studies, a DNA fragment corresponding to an N-terminal deletion mutant (residues 3596–4072) was incorporated into the same expression vector using the same method. In addition, to investigate the domains related to substrate recognition and binding, DNA fragments expressing truncated forms of *Vv*RRSP, namely residues 3610–4072, 3672–4072, 3737–4072, 3580–3855, and 3856–4072, were subcloned into the pHis-Parallel1 expression vector. For analysis of localization and cytotoxicity, a DNA fragment encoding residues 3580–4089 was amplified and inserted into the NheI and ApaI sites of the pAcGFP vector for expression of C-terminal GFP fusion proteins. Several plasmids expressing mutated or truncated forms of *Vv*RRSP fused with a C-terminal GFP, including RRSP_E3900L_, RRSP_E3930L_, RRSP_H3902L_, and RRSP_H4030L_, or residues 3580–3671, 3672–4089, 3610–4089, and 3737–4089, were generated by site-directed mutagenesis or subcloning as described above. For analysis of endopeptidase activity against endogenous Ras in cells, DNA fragments encoding *Vv*RRSP residues 3580–4089 were amplified and inserted into the XbaI and XhoI sites of the pEXPR-IBA103 vector for expressing the RRSP protein with Strep-tag in the C terminus. Constructs for expressing RRSP_E3900L_, RRSP_E3930L_, RRSP_H3902L_, RRSP_H4030L_, 3580–3671, 3737–4089, and 3672–4089 were also generated as described above for pEXPR-IBA103-RRSP (residues 3580–4089). The *k-ras* gene encoding KRas was amplified by PCR using the pCMV-SPORT6-KRas construct obtained from the Korea Human Gene Bank (KHGB) as a template, and the resulting PCR products were subcloned into the NcoI and XhoI sites of the pHis-Parallel1 expression vector. The DNA fragment encoding Raf1 (residues 54–131) was cloned from pCMV-SPORT6-Raf1 obtained from KHGB into the BamHI and XhoI sites of the pHis-Parallel1 expression vector.

A chemically synthesized DNA fragment encoding the C1/C2 region of PMT (Integrated DNA Technologies, Coralville, IA) was also subcloned into the pHis-Parallel1 expression vector to investigate enzymatic activity. All primers used for cloning and mutagenesis are listed in Table S2, and all mutated and cloned plasmids were confirmed by DNA sequencing (Macrogen, Korea).

### Protein expression and purification

For structural determination, His_6_-tagged *Vv*RRSP (residues 3596–4072) was overexpressed in *E. coli* BL21-CodonPlus (DE3)-RIPL cells by induction with 0.5 mm isopropyl β-d-thiogalactopyranoside (IPTG) at 21 °C for 18 h. Cultured cells were harvested by centrifugation at 5000 × *g* for 10 min at 4 °C, resuspended in ice-cold buffer A (50 mm Tris-HCl (pH 7.5), 300 mm NaCl), and lysed with a high-pressure homogenizer (Nano DeBEE, B.E.E. International). Crude cell extracts were centrifuged at 25,000 × *g* at 4 °C for 1 h. The supernatant containing hexaHis-tagged RRSP (residues 3596–4072) was loaded onto a Ni-NTA–agarose column (Qiagen Venlo, The Netherlands) pre-equilibrated with buffer A. Nonspecific resin-bound proteins were washed out with buffer A supplemented with 30 mm imidazole, and hexaHis-tagged RRSP was eluted with buffer A containing 250 mm imidazole. The hexaHis tag was cleaved by incubating the eluted protein with rTEV protease (Gibco) at 4 °C overnight. After protein concentration using a centrifugal concentrator with a 3-kDa cutoff (Amicon Ultra-15; Merck Millipore Ltd., Burlington, MA), size-exclusion chromatography was performed using a Superdex 200 10/300 column (GE Healthcare). Fractions containing RRSP (residues 3596–4072) were pooled and loaded onto a second Ni-NTA– agarose column for further purification, and the flow-through containing the tag-free RRSP (residues 3596–4072) was concentrated to 15 mg/ml and stored at −80 °C after clarification by centrifugation at 16,000 × *g* for 30 min. Selenomethionine (SeMet)-substituted RRSP (residues 3596–4072) was expressed in *E. coli* B834 (DE3) cells (Merck Millipore Ltd.), a methionine auxotrophic strain, in minimal medium supplemented with 50 mg/ml SeMet and purified as described above for native RRSP (residues 3596–4072) in the presence of 5 mm methionine throughout all purification steps. RRSP_E3930L/H4030L_ mutants were also expressed and purified as described for RRSP (residues 3596–4072). Purified proteins include a four-residue cloning artifact (Gly–Ala–Met–Ala) at the N terminus. For biochemical assays, expression of variants, including mutated and truncated forms, was carried out as described for RRSP (residues 3596–4072), but purification involved only one-step affinity column chromatography. Purified variants were stored at −80 °C after clarification by centrifugation at 16,000 × *g* for 30 min. Expression of KRas was induced in *E. coli* BL21-CodonPlus (DE3)-RIPL cells by treatment with 0.5 mm IPTG at 21 °C for 18 h. Harvested cells were resuspended in ice-cold buffer A and lysed using a high-pressure homogenizer (Nano DeBEE, B.E.E.). The recombinant N-terminally His_6_-tagged KRas in the supernatant was purified by Ni-NTA affinity chromatography, and the buffer was exchanged to 50 mm Tris-HCl (pH 7.5) and 300 mm NaCl for *in vitro* Ras cleavage assays. KRas fused with GSH *S*-transferase (GST) was also expressed and purified for *in vitro* pulldown analysis. The homogeneity of proteins was assessed by 12% SDS-PAGE and visualized using Coomassie Blue staining. Raf1 (residues 54–131) was made similar to that of KRas for pulldown analysis.

### Size-exclusion chromatography

A Superdex 200 10/300 GL gel-filtration column (GE Healthcare) installed on a fast protein LC (FPLC) system (Bio-Rad) was equilibrated with 50 mm Tris-HCl (pH 7.5) and 300 mm NaCl at a flow rate of 0.35 ml min^−1^ at 4 °C. Purified RRSP (residues 3596–4072) was injected onto the column, and bovine serum albumin (BSA) was used as a molecular weight standard.

### Crystallization, X-ray diffraction, and structure determination

Initial crystallization screening was performed using the sitting-drop vapor diffusion method against commercially available sparse-matrix screening kits (Hampton Research), with drops consisting of 0.4 μl of protein (15 mg/ml) in buffer A and 0.4 μl of reservoir solution in 96-well MRC plates (Hampton Research). Initial crystals were produced in several different conditions. However, high-quality crystals appropriate for X-ray diffraction were only obtained after optimizing conditions, and 1.4 m ammonium sulfate, 0.1 m Tris-HCl (pH 8.0), and 12% glycerol proved optimal. Crystals of RRSP (residues 3596–4072) were frozen in cryoprotectant solution consisting of 1.6 m ammonium sulfate, 0.1 m Tris-HCl (pH 8.0), and 12% glycerol and exposed to X-rays in a nitrogen gas stream at −173 °C. Crystals diffracted X-rays to a resolution of 2.7 Å at beamline 5C of the Pohang Accelerator Laboratory (PAL) in Korea. Crystals of SeMet-substituted RRSP (residues 3596–4072) were grown and optimized under the same conditions, and single-wavelength anomalous diffraction data were collected at a resolution of 2.9 Å using the same beamline. Crystals of RRSP_E3930L/H4030L_ were grown in 0.2 m LiSO_4_, 28% PEG 4000, and 0.1 m Tris-HCl (pH 8.0), and X-ray diffraction data were also collected at beamline 5C of PAL to a resolution of 2.3 Å. All X-ray diffraction data were processed and scaled with the HKL2000 software package (47). The structure of SeMet-substituted RRSP (residues 3596–4072) was initially determined with the AutoSol phasing module utilizing the anomalous signals from selenium atoms, and automatic model building was performed using AutoBuild in the PHENIX software package (48). Initial model building of SeMet-substituted RRSP (residues 3596–4072) was manually performed with COOT (49). The structure of native RRSP (residues 3596–4072) was solved by molecular replacement in MOLREP using the partial model of SeMet-substituted RRSP (residues 3596–4072) as a template (50). COOT was used for model building, and refinement, including the translation-liberation-screw procedure, was carried out with REFMAC5 and PHENIX (48, 51). The native structure was re-refined using the higher resolution mutant structure for further improvement of structural quality. Residues 3988–3992 and 4070–4072 in molecule A, 3989–3992 and 4068–4072 in molecule B, 3988–3992 and 4067–4072 in molecule C, and 3988–3992 and 4067–4072 in molecule D are disordered and thus not included in the final model. The structure of RRSP_E3930L/H4030L_ was solved using the first model of native RRSP as a template and refined in the same way. Residues 4069–4072 in molecule A, 3988–3991 and 4067–4072 in molecule B, 3989–3993 and 4066–4072 in molecule C, and 3988–3993, and 4066–4072 in molecule D are disordered and not included in the final model of RRSP_E3930L/H4030L_. Crystallographic data are summarized in Table 1.

### In vitro Ras cleavage and pulldown assays

Purified KRas (0.5 μm) was incubated with RRSP or PMT at a ratio of 1:1 or 2:1 in 50 mm Tris-HCl (pH 7.5) and 300 mm NaCl. After incubating the KRas-RRSP or KRas-PMT mixture at 37 °C for 20 min, the reaction was stopped by addition of SDS-PAGE sample buffer. Proteins were separated on a 15% gel and visualized using Coomassie Blue staining. For analysis of inhibitors of RRSP activity, purified RRSP was preincubated with the indicated protease inhibitors at 37 °C for 30 min. After adding KRas, the mixture was further incubated at the same temperature for the indicated time. Samples were separated by SDS-PAGE and visualized by Coomassie Blue staining.

For pulldown assays, plasmids expressing KRas fused with GST were used as bait proteins, mixed with Raf1 (residues 54–131) or variants of RRSP, including mutated and truncated forms, and incubated with GSH-conjugated Sepharose beads (GE Healthcare) in 50 mm Tris-HCl (pH 7.5), 300 mm NaCl, 1 mm GTP, and 5 mm MgCl_2_ for 3 h at 4 °C. After intensive washing with incubation buffer, KRas fused with GST was eluted with the same buffer supplemented with 20 mm reduced GSH. RRSP variants eluted from the resin were analyzed by SDS-PAGE and visualized using Coomassie Blue staining.

### Transient transfection

HEK293T cells were grown to ∼70–80% confluency, and transfection was implemented using X-tremeGENE HP reagent (Roche Applied Sciences, Penzberg, Upper Bavaria, Germany) according to the manufacturer's instructions.

### Endogenous Ras cleavage assay

HEK293T cells, transfected with plasmids expressing RRSP or its variants, were harvested at 16 h after transfection and lysed in lysis buffer (0.5% Nonidet P-40, 150 mm NaCl, 10% glycerol, and 20 mm HEPES (pH 7.2)) supplemented with complete protease inhibitor mixture (Roche Applied Science). After 30 min of incubation on ice, cell lysates were centrifuged at 16,000 × *g* for 30 min. Supernatants were mixed with LDS-PAGE loading buffer (Life Technologies, Inc.); proteins were separated by SDS-PAGE and transferred onto a polyvinylidene fluoride membrane by the semi-dry blot method; and RRSP, Ras, and Rap1 proteins were detected by Western blotting using a chemiluminescence detection system (ThermoFisher Scientific, Waltham, MA). To detect Strep-tagged RRSP and its variants, a horseradish peroxidase (HRP)-conjugated mouse anti-Strep antibody (IBA Lifesciences, Göttingen, Germany) was used according to the manufacturer's protocol. To detect endogenous Ras, mouse monoclonal anti-Ras antibody (1:2000; Millipore) and HRP-conjugated anti-mouse IgG antibody (1:5000; Cell Signaling Technology, Danvers, MA) were used. To detect endogenous Rap1, mouse monoclonal anti-Rap1 antibody (1:1000; Santa Cruz Biotechnology, Dallas, TX) and HRP-conjugated anti-mouse IgG antibody were used. Actin was detected using an HRP-conjugated rabbit monoclonal anti-β-actin antibody (1:5000; Cell Signaling Technology). All experiments were repeated at least three times, and data are expressed as the mean ± S.D. using Prism (Version 6.0, GraphPad Software).

### Cell morphological analysis

HEK293T cells were transfected with plasmids expressing the indicated RRSP C-terminal GFP constructs. After 16 h, cell morphological changes were examined using a FLoid Cell Imaging Station (ThermoFisher Scientific).

### Confocal microscopy

HEK293T cells were seeded into μ-slide 8-well plates (Ibidi, Martinsried, Germany) and transfected with plasmids expressing RRSP (residues 3580–4089) or its variants tagged with GFP at the C terminus. After 16 h, cells were fixed in 4% paraformaldehyde in phosphate-buffered saline (PBS) at 25 °C for 10 min. Fixed cells were washed with PBS and permeabilized with 100% methanol for 10 min at −20 °C. After further washing with PBS, cells were blocked with 2% BSA in PBS at 25 °C for 1 h and then incubated with Pan-Ras antibody (1:100; Millipore) overnight at 4 °C. After washing twice with PBS containing 0.1% Triton X-100 (PBS-T) and once with PBS, cells were incubated with anti-mouse IgG secondary antibody Alexa Fluor 568 (1:150; Invitrogen, Waltham, MA) at 25 °C for 1 h. After washing three times, nuclei were stained with Hoechst 33342 (ThermoFisher Scientific) at 25 °C for 10 min and further washed with PBS-T. To visualize actin filaments, RRSP-transfected cells were stained with rhodamine-phalloidin (1:1000; ThermoFisher Scientific) at 25 °C for 20 min. Mounting medium was applied to stained cells, and images were acquired using a Nikon laser-scanning confocal microscope (C2plus) and analyzed using NIS-Elements software.

### Circular dichroism (CD) spectroscopy

CD spectroscopy was used to compare the structural conformation of RRSP WT and its mutants. Pure protein (0.2 mg ml^−1^) in 50 mm Tris-HCl (pH 7.5) and 300 mm NaCl was subjected to far-UV CD measurements at 20 °C using a 1-mm path length quartz cuvette in a Jasco J-815 CD spectrometer (Jasco, Tokyo, Japan). CD spectra were acquired over the wavelength range of 200–260 nm and converted into mean residue ellipticity (degree cm^2^ dmol^−1^). Blank spectra obtained using buffer without protein were subtracted.

### PDB codes

The atomic coordinates and structure factor amplitudes of WT RRSP and the RRSPE_3930L/H4030L_ mutant have been deposited in the PDB (52) under accession codes 6A8J and 6A7H, respectively.

## Author contributions

S. Y. J., J. H., and M. H. K. conceptualization; S. Y. J., J. H., B. S. K., and E.-Y. L. data curation; S. Y. J., J. H., B. S. K., E.-Y. L., and M. H. K. formal analysis; S. Y. J., J. H., B. S. K., E.-Y. L., B.-H. O., and M. H. K. validation; S. Y. J., J. H., B. S. K., and E.-Y. L. investigation; S. Y. J., J. H., and M. H. K. visualization; S. Y. J., J. H., and E.-Y. L. methodology; J. H. writing-original draft; B.-H. O. and M. H. K. writing-review and editing; M. H. K. supervision; M. H. K. funding acquisition; M. H. K. project administration.

## Supplementary Material

Supporting Information

## Footnotes

5 The abbreviations used are:

MARTX: multifunctional autoprocessing repeats-in-toxin
RRSP: Ras/Rap1-specific endopeptidase
MLD: membrane localization domain
HRP: horseradish peroxidase
PMT: *P. multocida* toxin
PDB: Protein Data Bank
RMSD: root mean square deviation
rTEV: recombinant tobacco etch virus
Ni-NTA: nickel-nitrilotriacetic acid
IPTG: isopropyl β-d-thiogalactopyranoside
Ere: erythromycin esterase
ERK: extracellular signal-regulated kinase
SeMet: selenomethionine
GST: glutathione *S*-transferase.
