# Supplementary material for: Structural basis of inactivation of Ras and Rap1 small GTPases by Ras/Rap1-specific endopeptidase from the sepsis-causing pathogen Vibrio vulnificus
Source: J Biol Chem. 2018 Oct 3;293(47):18110–22. doi: 10.1074/jbc.RA118.004857 (PMC6254334; doi:10.1074/jbc.RA118.004857)
Supplement: Supporting Information [file supp_293_47_18110__index.html]

Structural basis of inactivation of Ras and Rap1 small GTPases by Ras/Rap1-specific endopeptidase from the sepsis-causing pathogen Vibrio vulnificus — Structure of Ras/Rap1-specific endopeptidase — Supporting Information 

# Structural basis of inactivation of Ras and Rap1 small GTPases by Ras/Rap1-specific endopeptidase from the sepsis-causing pathogen *Vibrio vulnificus*

## Supporting Information

- Supporting Information -

  Supplemental tables and figure legends
