# Supplementary material for: Structural basis of inactivation of Ras and Rap1 small GTPases by Ras/Rap1-specific endopeptidase from the sepsis-causing pathogen Vibrio vulnificus
Source: J Biol Chem. 2018 Oct 3;293(47):18110–22. doi: 10.1074/jbc.RA118.004857 (PMC6254334; doi:10.1074/jbc.RA118.004857)
Supplement: Supporting Information [file supp_RA118.004857_supplemental-data.pdf]

Supplemental Information

Structural basis of inactivation of Ras and Rap1 small GTPases by Ras/Rap1-specific endopeptidase from the sepsis-causing pathogen *Vibrio vulnificus*

Song Yee Jang[1,2§], Jungwon Hwang[2§*], Byoung Sik Kim[2,3], Eun-Young Lee[2], Byung-Ha Oh[1*], and Myung Hee Kim[2*]

From the [1]Department of Biological Sciences, Korea Advanced Institute of Science and Technology, Daejeon 34141, the [2]Infection and Immunity Research Laboratory, Metabolic Regulation Research Center, Korea Research Institute of Bioscience and Biotechnology, Daejeon 34141, and the [3]Department of Food Science and Engineering, Ewha Womans University, Seoul 03760, Korea

Running title: Structure of Ras/Rap1-specific endopeptidase

[§]These authors contributed equally to this work.

*To whom correspondence should be addressed: Jungwon Hwang, Infection and Immunity Research Laboratory, Korea Research Institute of Bioscience and Biotechnology, Daejeon 34141, Korea. Tel: +82-42-879-8223, E-mail: jwhwang@kribb.re.kr; Byung-Ha Oh, Department of Biological Sciences, Korea Advanced Institute of Science and Technology, Daejeon 34141, Korea. Tel: +82-42-350-2648, E-mail: bhoh@kaist.ac.kr; Myung Hee Kim, Infection and Immunity Research Laboratory, Korea Research Institute of Bioscience and Biotechnology, Daejeon 34141, Korea. Tel: +82-42-879-8219, E-mail: mhk8n@kribb.re.kr

Table S1. Bacterial strains and plasmids used in this study

Strain/plasmid	Relevant genotype/phenotype/characteristics	Sources
Escherichia coli		
DH5α	*F– Φ80lacZΔM15 Δ(lacZYA-argF) U169 recA1 endA1 hsdR17 (r_{K}–, m_{K}+) phoA supE44 λ– thi-1 gyrA96 relA1*	Invitrogen
BL21-CodonPlus (DE3)-RIPL	F- *ompT hsdS* (r_B^- m_B^-) *dcm*+ Tet^R gal λ (DE3) *endA* Hte [*argU proL* Cam^R] [*argU ileY leuW* Sm/$Spec^R$]	Agilent
B834 (DE3)	F- *ompT hsdSB* ($r_B^-m_B^-$) *gal dcm met* (DE3)	Novagen
**Plasmids**		
pHis-Parallel1 (pHisP)	Amp^R, a derivative of pFastBac-HTa (Nde) with a polylinker of pET22B, AF097413	(1)
pHisP_RRSP$_{3596-4072}$	Amp^R, wild-type RRSP (3596-4072, NcoI/XhoI) in pHis-Parallel1	This study
pHisP_RRSP$_{3580-4072}$	Amp^R, wild-type RRSP (3580-4072, NcoI/XhoI) in pHis-Parallel1	This study
pHisP_RRSP$_{E3900L}$	Amp^R, (3580-4072, NcoI/XhoI, E3900L) in pHis-Parallel1	This study
pHisP_RRSP$_{H3902L}$	Amp^R, (3580-4072, NcoI/XhoI, H3902L) in pHis-Parallel1	This study
pHisP_RRSP$_{Y3723A}$	Amp^R, (3580-4072, NcoI/XhoI, Y3723A) in pHis-Parallel1	This study
pHisP_RRSP$_{L3724A}$	Amp^R, (3580-4072, NcoI/XhoI, L3724A) in pHis-Parallel1	This study
pHisP_RRSP$_{E3930L}$	Amp^R, (3580-4072, NcoI/XhoI, E3930L) in pHis-Parallel1	This study
pHisP_RRSP$_{R3988E}$	Amp^R, (3580-4072, NcoI/XhoI, R3988E) in pHis-Parallel1	This study
pHisP_RRSP$_{H4030L}$	Amp^R, (3580-4072, NcoI/XhoI, H4030L) in pHis-Parallel1	This study
pHisP_RRSP$_{R4001F}$	Amp^R, (3580-4072, NcoI/XhoI, R4001F) in pHis-Parallel1	This study
pHisP_RRSP$_{3610-4072}$	Amp^R, wild-type RRSP (3610-4072, NcoI/XhoI) in pHis-Parallel1	This study
pHisP_RRSP$_{3672-4072}$	Amp^R, wild-type RRSP (3672-4072, NcoI/XhoI) in pHis-Parallel1	This study
pHisP_RRSP$_{3737-4072}$	Amp^R, wild-type RRSP (3737-4072, NcoI/XhoI) in pHis-Parallel1	This study
pHisP_RRSP$_{3581-3855}$	Amp^R, wild-type RRSP (3580-3855, NcoI/XhoI) in pHis-Parallel1	This study
pHisP_RRSP$_{3856-4072}$	Amp^R, wild-type RRSP (3856-4072, NcoI/XhoI) in pHis-Parallel1	This study
pHisP_PMT$_{569-1104}$	Amp^R, wild-type PMT (569-1104, NcoI/XhoI) in pHis-Parallel1	This study
pHisP_KRas$_{1-188}$	Amp^R, wild-type KRas (1-188, NcoI/XhoI) in pHis-Parallel1	This study
pHisP_Raf$_{54-131}$	Amp^R, Raf (54-131, BamHI/XhoI) in pHis-Parallel1	This study
pAcGFP_N1	Km^R, mammalian cell vector expressing GFP in the C-terminus of target protein	This study
pAcGFP_RRSP	Km^R, wild-type RRSP (3580-4089, NheI/ApaI) in pAcGFP_N1	This study
pAcGFP_RRSP$_{E3900L}$	Km^R, RRSP (3580-4089, NheI/ ApaI, E3900L) in pAcGFP_N1	This study
pAcGFP_RRSP$_{H3902L}$	Km^R, RRSP (3580-4089, NheI/ ApaI, H3902L) in pAcGFP_N1	This study
pAcGFP_RRSP$_{E3930L}$	Km^R, RRSP (3580-4089, NheI/ ApaI, E3930L) in pAcGFP_N1	This study
pAcGFP_RRSP$_{H4030L}$	Km^R, RRSP (3580-4089, NheI/ ApaI, H4030L) in pAcGFP_N1	This study
pAcGFP_RRSP$_{3580-3671}$	Km^R, RRSP (3580-3671, NheI/ ApaI) in pAcGFP_N1	This study
pAcGFP_RRSP$_{3737-4089}$	Km^R, RRSP (3737-4089, NheI/ ApaI) in pAcGFP_N1	This study
pAcGFP_RRSP$_{3610-4089}$	Km^R, RRSP (3610-4089, NheI/ ApaI) in pAcGFP_N1	This study

pAcGFP_RRSP$_{3672-4089}$	Km^R, RRSP (3672-4089, NheI/ ApaI) in pAcGFP_N1	This study
pEXPR_IBA103	Amp^R, mammalian cell vector expressing Strep-tag in the C-terminus of target protein	This study
pEXPR_RRSP	Amp^R, wild-type RRSP (3580-4089, XbaI/XhoI) in pEXPR_IBA103	This study
pEXPR_RRSP$_{E3900L}$	Amp^R, RRSP (3580-4089, XbaI/XhoI, E3900L) in pEXPR_IBA103	This study
pEXPR_RRSP$_{H3902L}$	Amp^R, RRSP (3580-4089, XbaI/XhoI, H3902L) in pEXPR_IBA103	This study
pEXPR_RRSP$_{E3930L}$	Amp^R, RRSP (3580-4089, XbaI/XhoI, E3930L) in pEXPR_IBA103	This study
pEXPR_RRSP$_{H4030L}$	Amp^R, RRSP (3580-4089, XbaI/XhoI, H4030L) in pEXPR_IBA103	This study
pEXPR_RRSP$_{3580-3671}$	Amp^R, RRSP (3580-3671, XbaI/XhoI) in pEXPR_IBA103	This study
pEXPR_RRSP$_{3672-4089}$	Amp^R, RRSP (3672-4089, XbaI/XhoI) in pEXPR_IBA103	This study
pEXPR_RRSP$_{3737-4089}$	Amp^R, RRSP (3737-4089, XbaI/XhoI) in pEXPR_IBA103	This study
pCMV-SPORT6-KRas	Amp^R, KRas (1-188) in pCMV-SPORT6	This study

Km^R, kanamycin resistance; Amp^R, ampicillin resistance.

Table S2. Oligonucleotides used for cloning and site-directed mutagenesis

Oligonucleotide	Sequence (5' to 3') ¶	Use
(pHis-Parallel)		
RRSP$_{3596-4072}$	CATGCCATGGCCCAAGAGCTGAAAGAAAG	Cloning
RRSP$_{3580-4072}$	ACGTCCATGGATAAAACCAAGGTCGTG	Cloning
RRSP$_{E3900L}$	GTAAAGGCCTCTTGATTGGTCTAGTGCATGGCAGCGACGTC	Mutagenesis
RRSP$_{H3902L}$	CTTGATTGGTGAAGTGCTTGGCAGCGACGTCAAC	Mutagenesis
RRSP$_{Y3723A}$	GGCCAATGAAGACCCAGCCTTGGCACCGAAGCATG	Mutagenesis
RRSP$_{L3724A}$	CAATGAAGACCCATACGCGGCACCGAAGCATGGTTAC	Mutagenesis
RRSP$_{E3930L}$	GGGAGTCACAGTCATTGGCCTTTTGCATTTACGCTCAGACCTTGCGC	Mutagenesis
RRSP$_{R3988E}$	GCTGGATGCAAACAGCTCTGCGGAACCAAATGTTCAGGGAACAGAAC	Mutagenesis
RRSP$_{H4030L}$	GTTGCTATCTACGGTAAAGCGCTTTTGCAGTCTCACAAAGGGATTG	Mutagenesis
RRSP$_{R4001F}$	GGAACAGAACATGGTCTGATGTACTTTGCTGGTGCTGCGAACAACATTGCG	Mutagenesis
RRSP$_{3610-4072}$	CATGCCATGGGCGCATCCTACCAAGG	Cloning
RRSP$_{3672-4072}$	CATGCCATGGGTAAGATGCAAGTTGCC	Cloning
RRSP$_{3737-4072}$	CATGCCATGGACCTTGGTTTTGATGCGAAG	Cloning
RRSP$_{3580-3855}$	ACGTCCATGGATAAAACCAAGGTCGTG	Cloning
RRSP$_{3856-4072}$	CATGCCATGGATATTGACGCTTGGGATCG	Cloning
PMT$_{569-1104}$	CATGCCATGGTAAGCAATGCTAAACTATTAGG	Cloning
KRas$_{1-188}$	CATGCCATGGCCACTGAATATAAACTTGTGG	Cloning
Raf$_{54-131}$	CGGGCGGATCCCACAAGCAACACTATCCG	Cloning
(pAcGFP vector)		
RRSP$_{3580-4089}$	CTAGCTAGCGATGGGTGATAAAACCAAGGTCG	Cloning
RRSP$_{3580-3671}$	CTAGCTAGCGATGGGTGATAAAACCAAGGTCG	Cloning
RRSP$_{3672-4089}$	CTA GCTAGCGATGGGTAAGATGCAAGTTGCC	Cloning
RRSP$_{3610-4089}$	CTAGCTAGCGATGGGCGCATCCTACCAAGG	Cloning
RRSP$_{3737-4089}$	CTAGCTAGCGATGGACCTTGGTTTTGATGCG	Cloning
(pEXPR-IBA103 vector)		
RRSP$_{3580-4089}$	CTAGTCTAGACCCACAATGGGTGATAAAACCAAGGTCGTG	Cloning
RRSP$_{3580-3671}$	CTAGTCTAGACCCACAATGGGTGATAAAACCAAGGTCGTG	Cloning
RRSP$_{3672-4089}$	CTAGTCTAGAATGGGTAAGATGCAAGTTGCC	Cloning
RRSP$_{3737-4089}$	CTAGTCTAGAATGGACCTTGGTTTTGATGCG	Cloning

¶Regions of oligonucleotides not complementary to the corresponding genes are underlined.

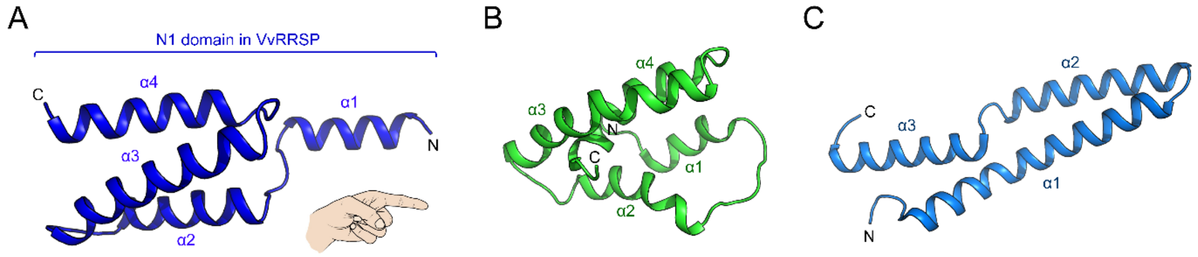

Figure S1. Structural comparison of N1 domains. *A*, the structure of the N1 domain in *Vv*RRSP shown in cartoon representation. Each helix is indicated. *B*, the structure of an isolated MLD (PDB ID 2N9W) determined by nuclear magnetic resonance (NMR). *C*, the structure of an isolated MLD (PDB ID 4ERR) determined by X-ray crystallography.

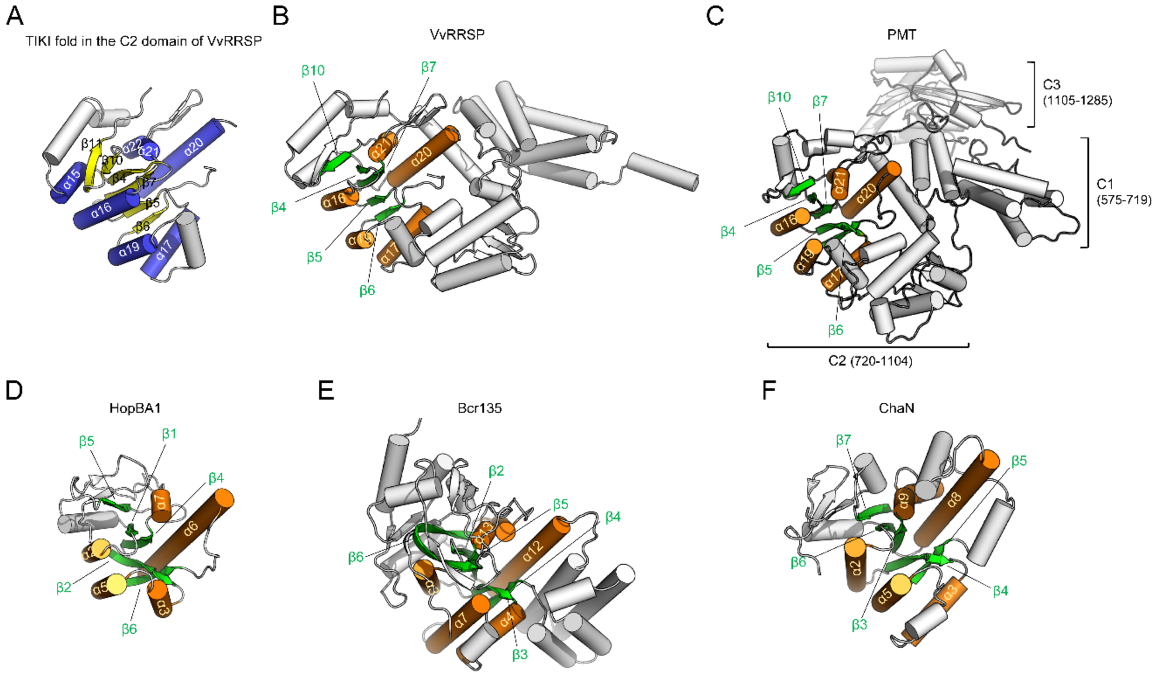

Figure S2. Structural comparison of *Vv*RRSP and TIKI fold superfamily proteins. *A*, the TIKI fold in the C2 domain of *Vv*RRSP. Central β-strands and neighboring helices in the TIKI fold of the C2 domain are colored yellow and blue, respectively. *B–F*, structures of TIKI fold-containing superfamily proteins *Vv*RRSP (*B*), PMT (PDB ID 2EBF; *C*), HopBA1 (PDB ID 5T09; *D*), Bcr135 (PDB ID 3B55; *E*), and ChaN (PDB ID 2G5G; *F*). The parallel β-sheet core (green) and neighboring helices (orange) in each structure are indicated. C1, C2, and C3 domains in PMT are indicated in *C*.

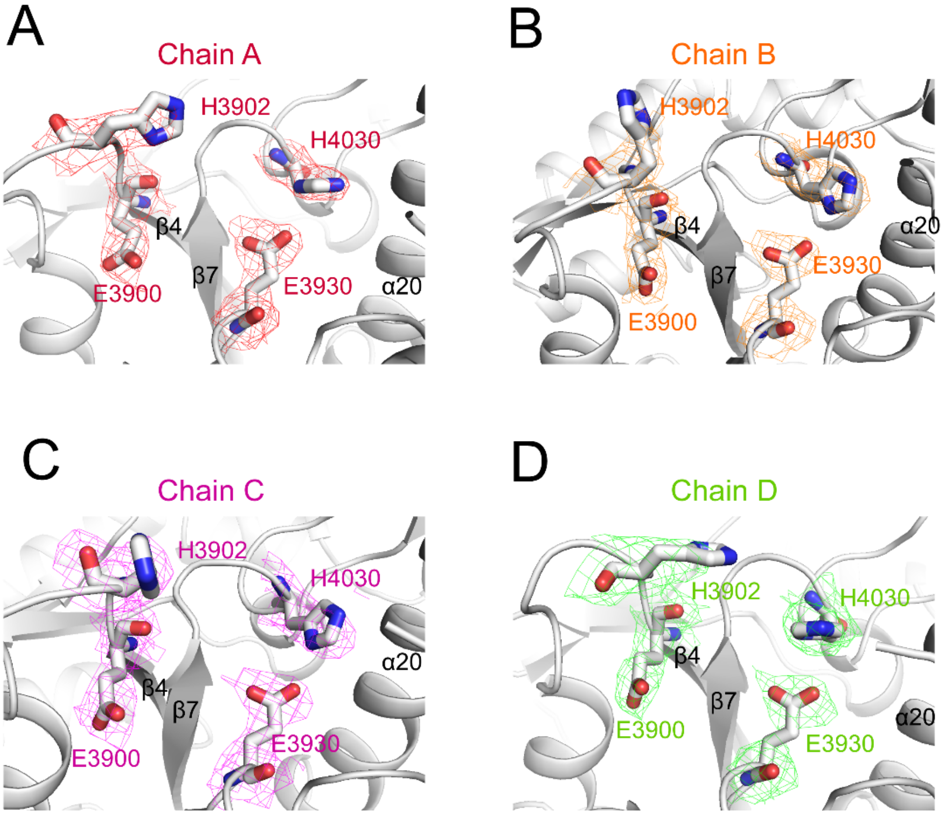

Figure S3. 2Fo-Fc electron density maps showing catalytic functional residues of WT RRSP. The electron density maps are contoured at 1.0 σ and colored red for Chain A (*A*), orange for Chain B (*B*), magenta for Chain C (*C*), and green for Chain D (*D*).

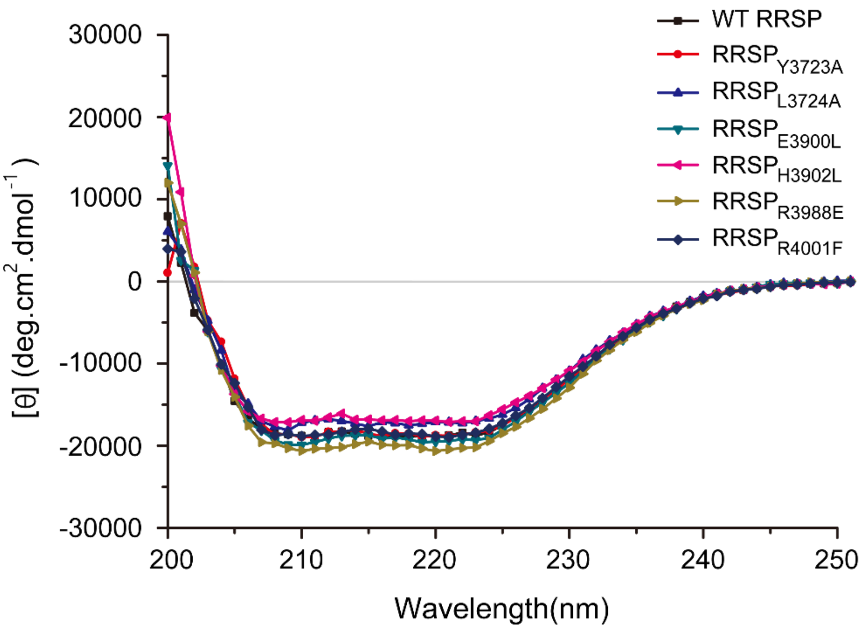

Figure S4. Circular dichroism (CD) spectroscopy analysis of WT RRSP and its mutant proteins. Typical far-UV CD spectra of WT RRSP and it mutants are shown.

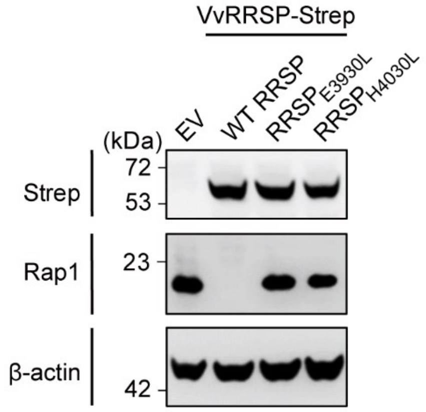

Figure S5. Endogenous Rap1 processing activity of *Vv*RRSP. HEK293T cells transfected with plasmids expressing the indicated Strep-tagged proteins were lysed and immunoblotted with anti-Strep or anti-Rap1 antibody. Actin was used as a loading control. EV, empty vector.

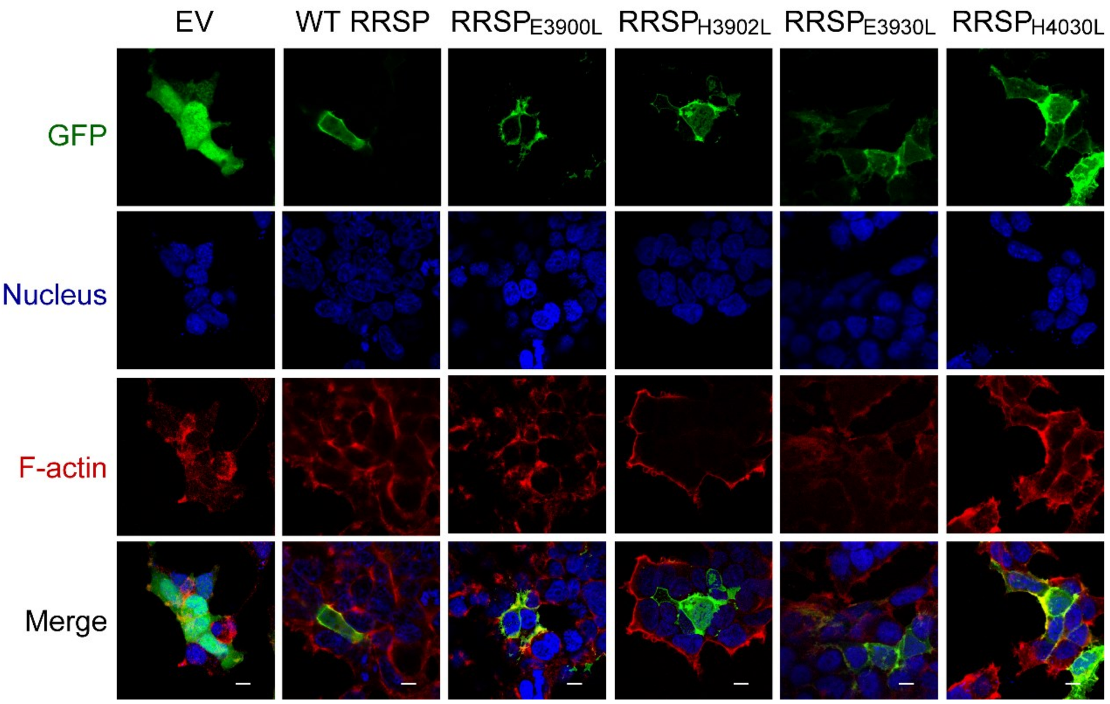

Figure S6. Localization of *Vv*RRSP in cells. HEK293T cells were transfected with GFP fusion plasmids expressing the indicated proteins for 16 h. Actin filaments (F-actin) were stained with rhodamine-phalloidin (red), and nuclei were stained with Hoechst (blue). *Scale bars* = 10 μm.

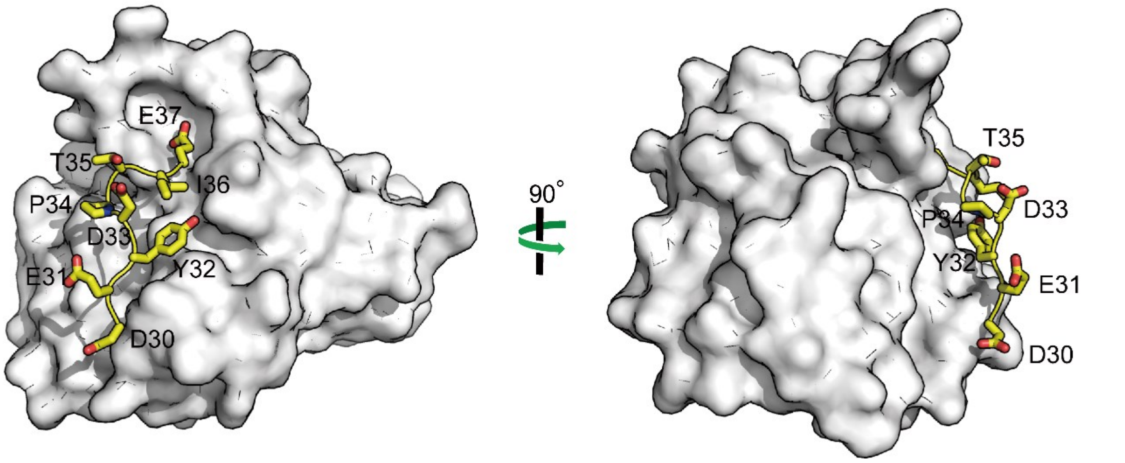

Figure S7. Residues in the Switch I region of KRas. Residues in the Switch I region are displayed in stick representation (yellow) on the structure of KRas shown in surface representation (gray).

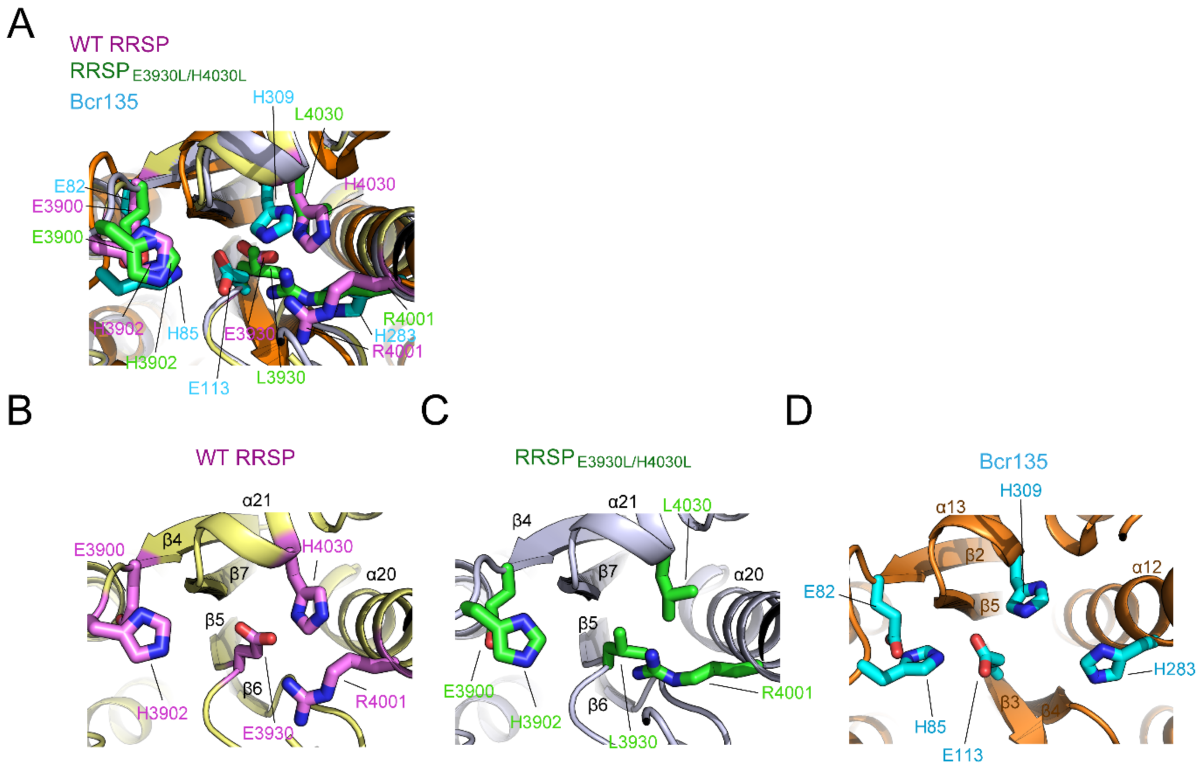

Figure S8. The active sites of WT RRSP and the RRSP$_{E3930L/H4030L}$ and Bcr135 mutants. *A*, superimposition of WT RRSP, RRSP $_{E3930L/H4030L}$, and Bcr135. *B–D*, functional residues in the active sites of WT RRSP, RRSP $_{E3930L/H4030L}$, and Bcr135 are shown in magenta, green, and cyan, respectively.

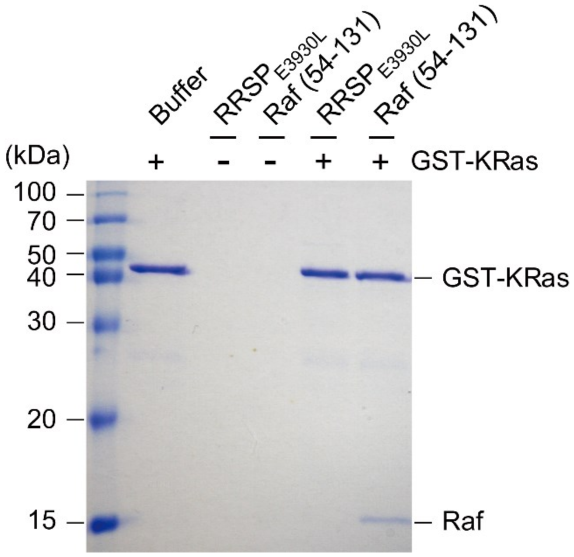

Figure S9. Pull-down assays using GST-fused KRas. There is clearly no interaction of $RRSP_{E3930L}$ with GST-fused KRas. His-tagged Raf1 (residues 54–131) that interacts with GST-fused KRas was used as a positive control.

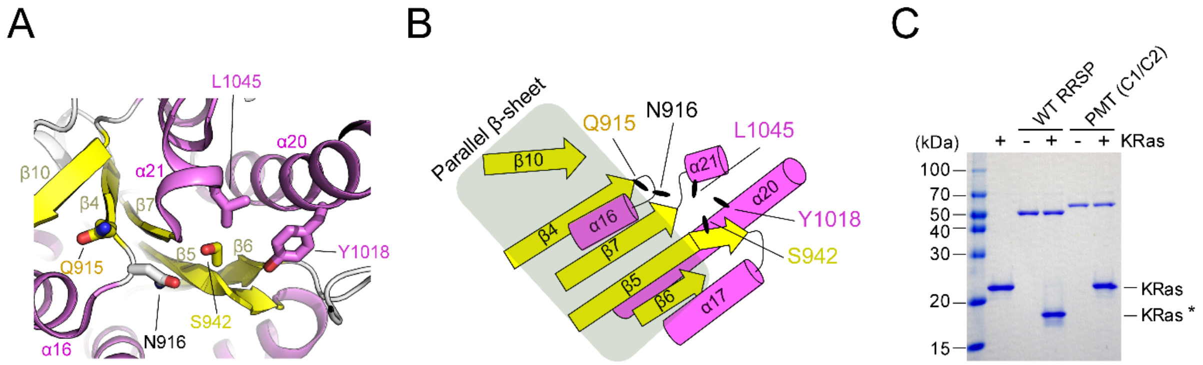

Figure S10. The conserved TIKI fold structure in PMT. *A*, the TIKI fold in PMT showing residues corresponding to functional residues in the C2 catalytic domain of *Vv*RRSP. The parallel β-sheet core and neighboring helices are colored yellow and magenta, respectively. *B*, topology of the β-sheet core and neighboring helices in PMT. Residues in PMT corresponding to functional residues in RRSP are indicated and shown as elliptical shapes (black). *C*, *in vitro* KRas cleavage assay of PMT demonstrating a lack of KRas cleavage activity. KRas* indicates cleaved KRas.

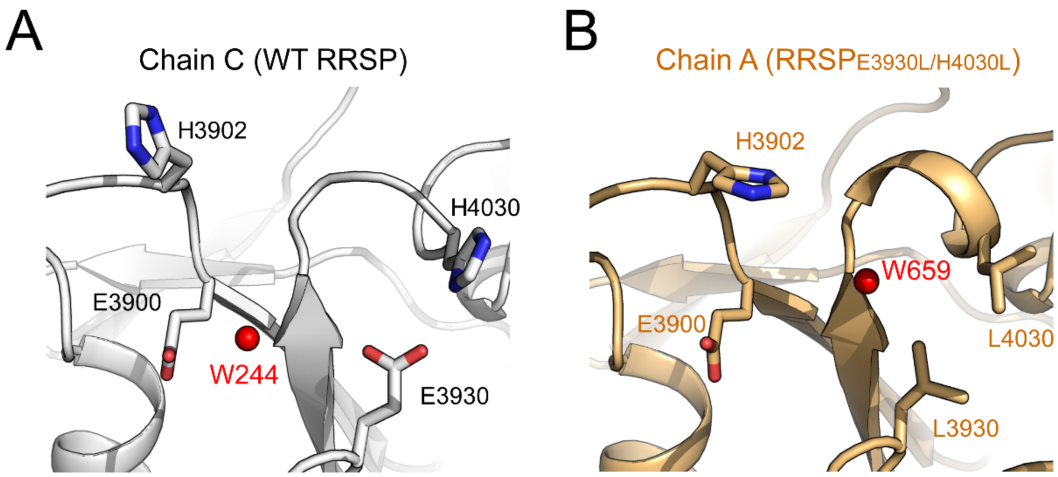

Figure S11. The catalytic site in *Vv*RRSP. *A* and *B*, structures of active sites in C2 domains in WT RRSP (*A*) and the $RRSP_{E3930L/H4030L}$ mutant (*B*) colored white and orange, respectively. The water molecule is shown as a red sphere in each active site.

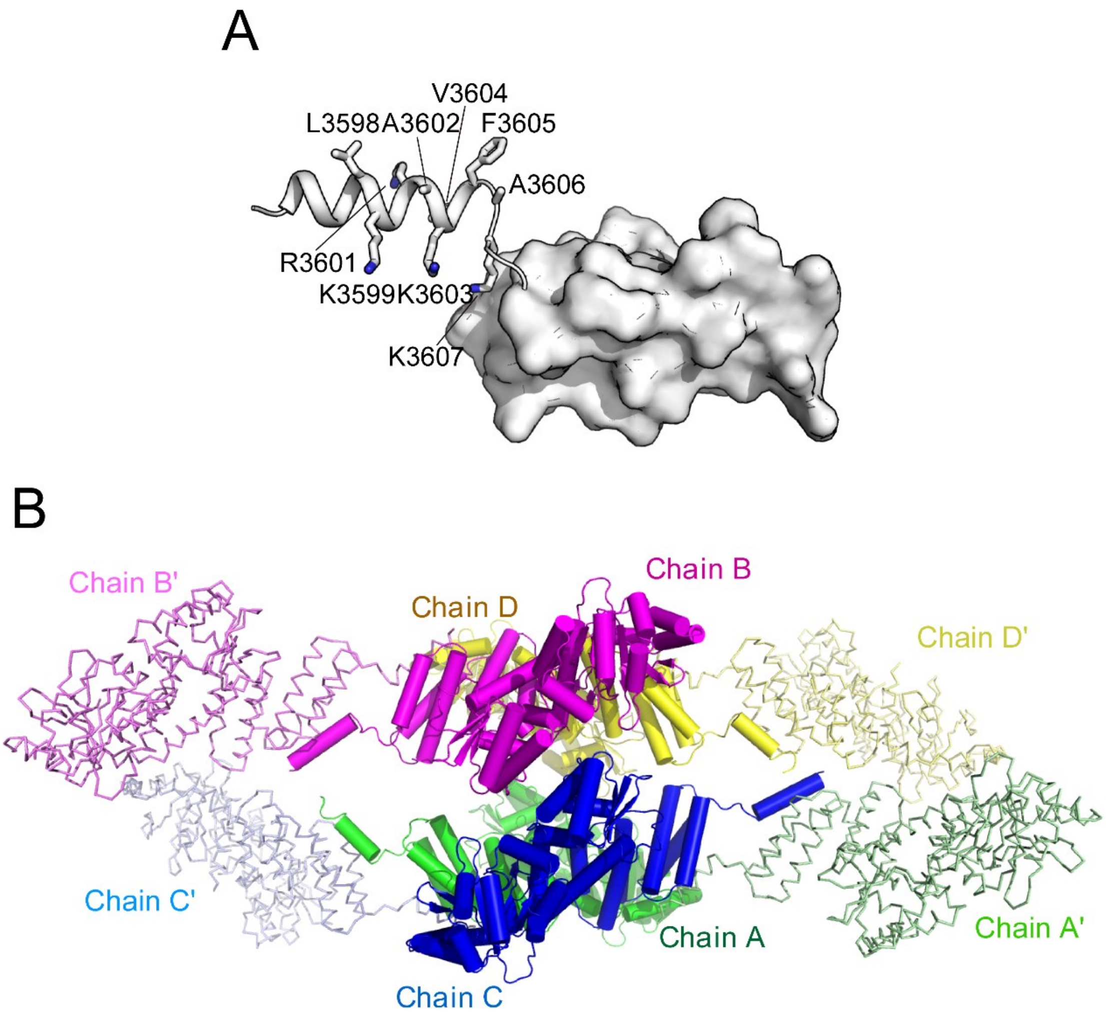

Figure S12. Helix α1 in *Vv*RRSP. *A*, hydrophobic and positively charged residues on helix α1 are displayed in stick representation, and the remaining three-helix bundle in the N1 domain is shown in surface representation (gray). *B*, crystal packing of WT RRSP is displayed in cartoon representation. The four RRSP molecules in the asymmetric unit are colored green, magenta, blue, and yellow, respectively. Neighboring chains in the other asymmetric units are shown in ribbon representation.

Reference

1. Sheffield, P., Garrard, S., and Derewenda, Z. (1999) Overcoming expression and purification problems of RhoGDI using a family of "parallel" expression vectors. *Protein expression and purification* **15**, 34-39
